# Genomic diversity of *Mycobacterium avium* subsp. *paratuberculosis*: pangenomic approach for highlighting unique genomic features with newly constructed complete genomes

**DOI:** 10.1186/s13567-021-00905-1

**Published:** 2021-03-18

**Authors:** Jaewon Lim, Hong-Tae Park, Seyoung Ko, Hyun-Eui Park, Gyumin Lee, Suji Kim, Min-Kyoung Shin, Han Sang Yoo, Donghyuk Kim

**Affiliations:** 1 School of Life Sciences, Ulsan National Institute of Science and Technology (UNIST), Ulsan, Korea; 2 Department of Infectious Disease, College of Veterinary Medicine, Seoul National University, Seoul, Korea; 3 Department of Microbiology, Research Institute of Life Science, College of Medicine, Gyeongsang National University, Jinju, Korea; 4 School of Energy and Chemical Engineering, Ulsan National Institute of Science and Technology (UNIST), Ulsan, Korea; 5 Bio-MAX/N-Bio Institute, Seoul National University, Seoul, 08826 Korea

**Keywords:** *Mycobacterium avium* subsp. *paratuberculosis*, Whole genome sequence, Pangenome, Molecular epidemiology, Genetic polymorphism

## Abstract

*Mycobacterium avium* subsp. *paratuberculosis* (MAP) is a causative agent of Johne’s disease, which is a chronic granulomatous enteropathy in ruminants. Determining the genetic diversity of MAP is necessary to understand the epidemiology and biology of MAP, as well as establishing disease control strategies. In the present study, whole genome-based alignment and comparative analysis were performed using 40 publicly available MAP genomes, including newly sequenced Korean isolates. First, whole genome-based alignment was employed to identify new genomic structures in MAP genomes. Second, the genomic diversity of the MAP population was described by pangenome analysis. A phylogenetic tree based on the core genome and pangenome showed that the MAP was differentiated into two major types (C- and S-type), which was in keeping with the findings of previous studies. However, B-type strains were discriminated from C-type strains. Finally, functional analysis of the pangenome was performed using three virulence factor databases (i.e., PATRIC, VFDB, and Victors) to predict the phenotypic diversity of MAP in terms of pathogenicity. Based on the results of the pangenome analysis, we developed a real-time PCR technique to distinguish among S-, B- and C-type strains. In conclusion, the results of our study suggest that the phenotypic differences between MAP strains can be explained by their genetic polymorphisms. These results may help to elucidate the diversity of MAP, extending from genomic features to phenotypic traits.

## Introduction

*Mycobacterium avium* subsp. *paratuberculosis* (MAP) is a causative agent of Johne’s disease, which is a chronic granulomatous enteropathy in ruminants. MAP infection is characterized by chronic diarrhea, progressive wasting, and eventual death. MAP infection is also economically important because infected individuals exhibit weight loss and reduced milk production [1]. The disease has been observed primarily in ruminants (e.g., cattle, sheep, goats, and deer) and various other nonruminant animals worldwide [2]. In addition, the association of MAP with Crohn’s disease, which is a type of chronic inflammatory bowel disease in humans, has been noted in numerous studies [3].

Determining the genetic diversity of MAP is necessary to understand the epidemiology and biology of MAP, as well as establishing disease control strategies [4, 5]. MAP strains are differentiated into two major groups, known as “Cattle type” or “C-type” and “Sheep type” or “S-type”, which are named after the host species of first isolation [6]. An additional group of strains, known as “Bison type” or “B-type”, were first differentiated based on a single nucleotide polymorphism (SNP) at the 223-bp position of IS*1311* [7]. Some molecular typing techniques have been developed, such as variable-number tandem repeats of mycobacterial interspersed repetitive units (MIRU-VNTR) and multilocus short sequence repeat (MLSSR), to elucidate the genetic diversity and investigate the molecular epidemiology of MAP strains [8, 9]. More recently, phylogenetic analysis using SNPs based on whole genome sequencing (WGS) was determined to provide greater resolution between isolates [4, 10]. Despite the notable advances made with molecular typing of epidemiological traits, strain-specific differences in the virulence and pathogenicity of MAP have not been thoroughly investigated to date [11]. Research attempting to identify correlations between genetic diversity and phenotypic differences has primarily focused on the major strain types [12–15].

The increased accuracy and lower cost of WGS has facilitated efforts to accumulate WGS data, which enabled researchers to perform comparative genomic analyses. Comparative genomics is a widely employed analytical method to identify differences or similarities among bacteria [16]. Among the various analysis techniques, pangenome analysis is a powerful tool for comparing bacterial strains. Pangenome analysis can identify the genes that are shared in groups or the genes that exhibit different appearances in certain groups based on clustering of the sequences of genes. Through this analysis, we can provide insight into the evolution of the species. The genome to be compared can be differentiated into core, accessory, and unique genomes according to similarity. The “core genome” is a group of genes shared in all strains, the “accessory genome” contains shell genes present in more than two strains but not in all strains, and the “unique genome” contains genes specific to single strains. The core genes are responsible for the major phenotypic traits of the species and its survival, while the accessory genes and unique genes are generally related to supplementary biochemical pathways and functions that may provide selective advantages, such as ecological adaptation, virulence mechanisms, antibiotic resistance, or colonization of a new host [17].

Although there are many studies based on the whole genome of MAP, more whole genome data of various strains of MAP worldwide are still needed. In particular, there is no complete level of genome sequences for strains known as B-type. Therefore, in this study, complete genome sequencing was performed on B-type strains, which account for a large proportion of the strains detected in Korea, and C-type strains isolated in the same region. Subsequently, the standard strain of MAP and the newly sequenced genome derived from Korea were compared and analyzed using the genome alignment technique. New large gaps and novel tandem repeats (TRs) were observed from the whole genome alignment between the Korean strains and reference genome K-10. Furthermore, pangenome analysis was performed using the whole genome database of MAP to describe the genomic features of the MAP with respect to genetic diversity, especially in virulence-related genes. Through the analysis, we also attempted to define the links between population structure and pathogenicity. Pangenome analysis was performed with 40 MAP genomes and confirmed that MAP has a well-conserved genome, even though there were a large number of S-type-specific genes. The virulence factors (VFs) were searched in the pangenome with three different databases. It was confirmed that most of the known VFs were conserved in all genomes. Finally, we designed a real-time PCR technique with a novel biomarker gene identified in this study to efficiently distinguish the type of MAP strains.

## Materials and methods

### Bacterial genomes of Korean MAP (MAPK) strains

**Figure 1:**
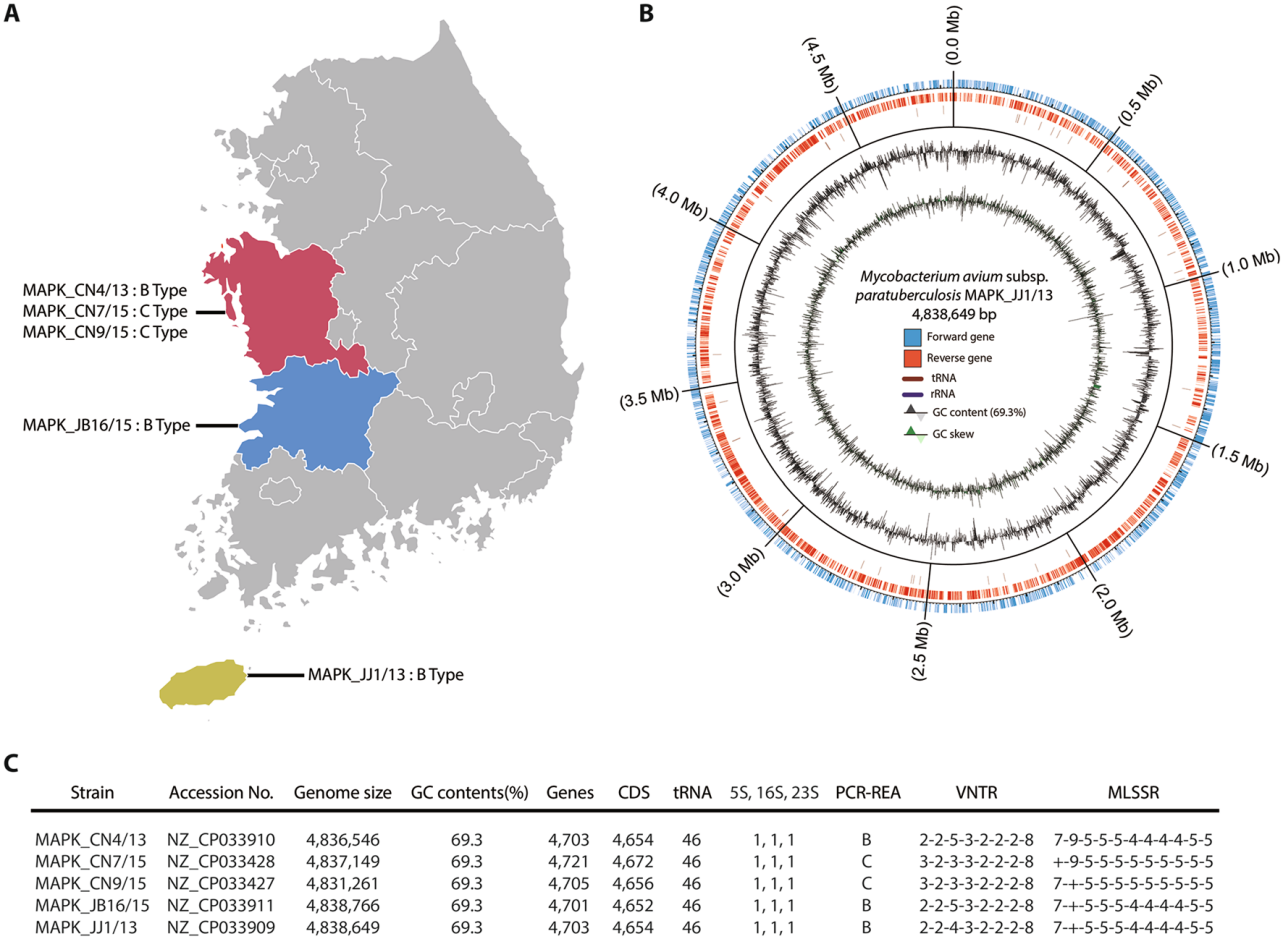
**Genome reconstruction of five newly isolated** ***M. avium*** **subsp.** ***paratuberculosis*** **strains in Korea.** **A** Geographical locations for isolation of 5 MAP strains. **B** Circular visualization of genomic reconstruction for the representative strain MAPK_JJ1/13. **C** Genomic features and genotype characterization of 5 MAP strains.

In our previous study, 19 MAP isolates were obtained from 10 cattle herds in 5 provinces in Korea, and their genetic diversity was analyzed [18]. Genetic classification using IS*1311* PCR-REA, MIRU-VNTR, and MLSSR typing was employed to differentiate the isolates into 5 different groups. In this study, five isolates were selected for WGS based on genotyping results: three B-type isolates (MAPK_JJ1/13, MAPK_CN4/13 and MAPK_JB16/15, which were isolated from different provinces and differentiated into three different groups by the difference in locus 2 in MLSSR typing) and two C-type isolates (MAPK_CN7/15 and MAPK_CN9/15, which were isolated from a herd and showed differences at locus 1 and locus 2 in MLSSR typing) (Figure 1A).

### Genome sequencing

The library was constructed with SMRTbell™ Template Prep Kit 1.0 (PN 100-259-100) following the manufacturer’s instructions (Pacific Biosciences). The constructed library was validated by an Agilent 2100 Bioanalyzer. The SMRTbell library was sequenced using SMRT cells (Pacific Biosciences) using C4 chemistry (DNA sequencing Reagent 4.0), and 240 min movies were captured for each SMRT cell using the PacBio RS II (Pacific Biosciences) sequencing platform [19]. De novo assembly was conducted using the hierarchical genome assembly process (HGAP, Version 2.3) workflow [20], including consensus polishing with Quiver. The estimated average coverage of the five MAP strains was 84X-137X.

### Genome selection and reannotation

Whole-genome sequence data of five newly sequenced MAP strains and 35 other MAP strains were downloaded from NCBI, and reannotation was performed by using RAST [21]. Even though 50 MAP genomes were registered in the NCBI database, 40 annotated genomes were used for analysis, since 10 of them were excluded due to the low confidence of genomes (e.g., fragmented assembly, numerous frameshifted proteins, or untrustworthy as type).

### Genome-wide alignment of MAPK strains

Whole-genome sequence alignment of 14 MAP genomes was performed by the Mauve alignment tool (ver. 20150226) with default options of the progressiveMauve algorithm. Except for the JIII-386 strain, all MAP genomes were complete genomes. New large gaps and tandem repeats were discovered from this analysis, and the found information was confirmed by utilizing the NCBI database.

### In silico VNTR typing

Novel tandem repeats (TRs) identified in this study were further analyzed with 13 complete genome sequences of MAP available in NCBI. The genotype of each genome was verified by aligning the sequences of the TR region (TR type). The discriminatory index (DI) described by Hunter and Gaston [22] was used to calculate the discriminatory power of the in silico typing with novel TRs [22]. The DI was calculated using the following equation:

$$DI = 1 - \left[ {\frac{1}{{N\left( {N - 1} \right)}}\mathop \sum \limits_{j = 1}^{s} n_{j} \left( {n_{j} - 1} \right)} \right],$$

where *N* is the total number of isolates in the typing scheme, *s* is the total number of distinct types discriminated by each typing method, and n_*j*_ is the number of isolates belonging to the *j*th type.

### Pangenome analysis and phylogeny analysis

The Bacterial Pangenome Analysis (BPGA) Tool [17] was used as the analysis pipeline for both pangenome analyses and phylogenetic analysis. For the pangenome analysis, coding sequences (CDSs) with protein sequences were used. Each CDS information was extracted from the annotated genome in “fasta” format. The identity cutoff was 0.9 (90%) for the similarity calculation. For the phylogenetic analysis, MUSCLE [23], a built-in tool in BPGA, was used. The core-genome phylogeny was generated based on concatenated core gene alignment, and the binary panmatrix phylogeny was generated using a panmatrix (binary gene presence/absence (1/0) matrix).

### Functional analysis (COG, VF)

Functional analysis was performed based on the Basic Local Alignment Search Tool (BLAST) algorithm. USEARCH [24] was used to run the BLAST. Four databases were used for the analysis: Virulence Factors of Pathogenic Bacteria (VFDB) [25], Victors [26], and PATRIC [27] for virulence factors and Clusters of Orthologous Groups (COGs) [28] for functional analysis. The identity cutoff was 0.8 (80%) for both functional analyses.

### Genomic DNA extraction and PCR analysis

A total of 12 isolates were obtained from Korea (*n* = 9), Czech (*n* = 1), and *M. avium* subsp. *hominissuis* strain 104 and 101 (*n* = 2). Each isolate was cultured in 5 mL of Middlebrook 7H9 broth supplemented with Middlebrook OADC and mycobactin J (2 mg/L). After incubation at 37 °C for 6 weeks, the bacterial suspension was centrifuged at 3000 × *g* for 20 min, and the pellet was lysed in 1 mL of L6 lysis buffer (5.25 M GuSCN, 50 mM Tris–HCl at pH 6.4, 20 mM EDTA, 1.3% Triton X-100, distilled water). Subsequent processes were performed as previously described [29]. All PCRs were performed in a final reaction volume of 20 μL and contained 200 pg of DNA template, 10 μL of 2X THUNDERBIRD SYBR qPCR Mix (Toyobo Co., Ltd., Osaka, Japan), and 1 pg of each primer. PCR was performed by using an Applied Biosystems QuantStudio 3 Real-Time PCR system under the following conditions: initial denaturation (95 °C for 3 min); next, 40 cycles of denaturation (95 °C for 30 s), annealing (55 °C for 30 s), and extension (72 °C for 30 s).

## Results

### Genomic characterization of five newly isolated *M. avium* subsp. *paratuberculosis* strains in Korea

Genome sequencing was conducted with five *M. avium* subsp. *paratuberculosis* (MAP) strains that were isolated from Korea as previously described [18]. Three strains, MAPK_CN9, MAPK_CN7, and MAPK_CN4, were isolated from a farm in the Chungcheongnamdo region, and two other strains, MAPK_JB16 and MAPK_JJ1, were isolated from two farms in Jeollabukdo and Jejudo (Figure 1A). Genome reconstruction results were visualized in circular form (Figure 1B, Additional file 1). The average genome size of the strains was calculated as 4 836 474 bp, and their average GC content was 69.3% (Figure 1C). The number of predicted genes was 4707 on average, and among them, 4658 were coding sequences (CDSs).

**Figure 2:**
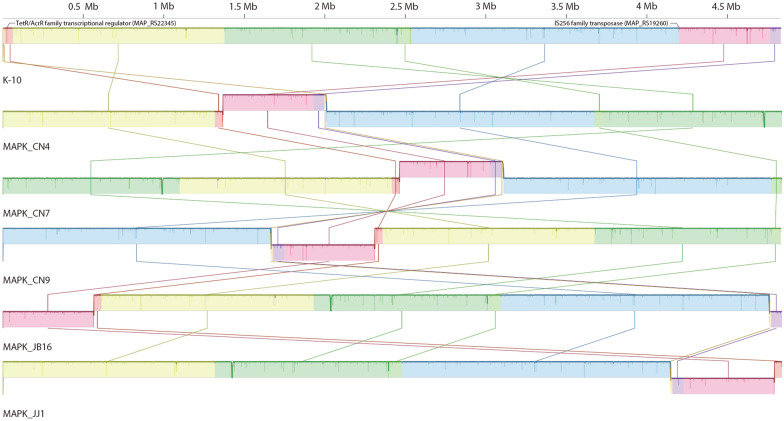
**Whole genome sequence alignment of five MAPK strains and reference strain K-10.** The genome inversion was observed from the result. The inverted genome positions were between two genes that were predicted as IS*256* family transposases (MAP_RS19260, 4.2 Mb in K10) and TetR/AcrR family transcriptional regulator (MAP_RS22345, 11 kb in K10).

**Figure 3:**
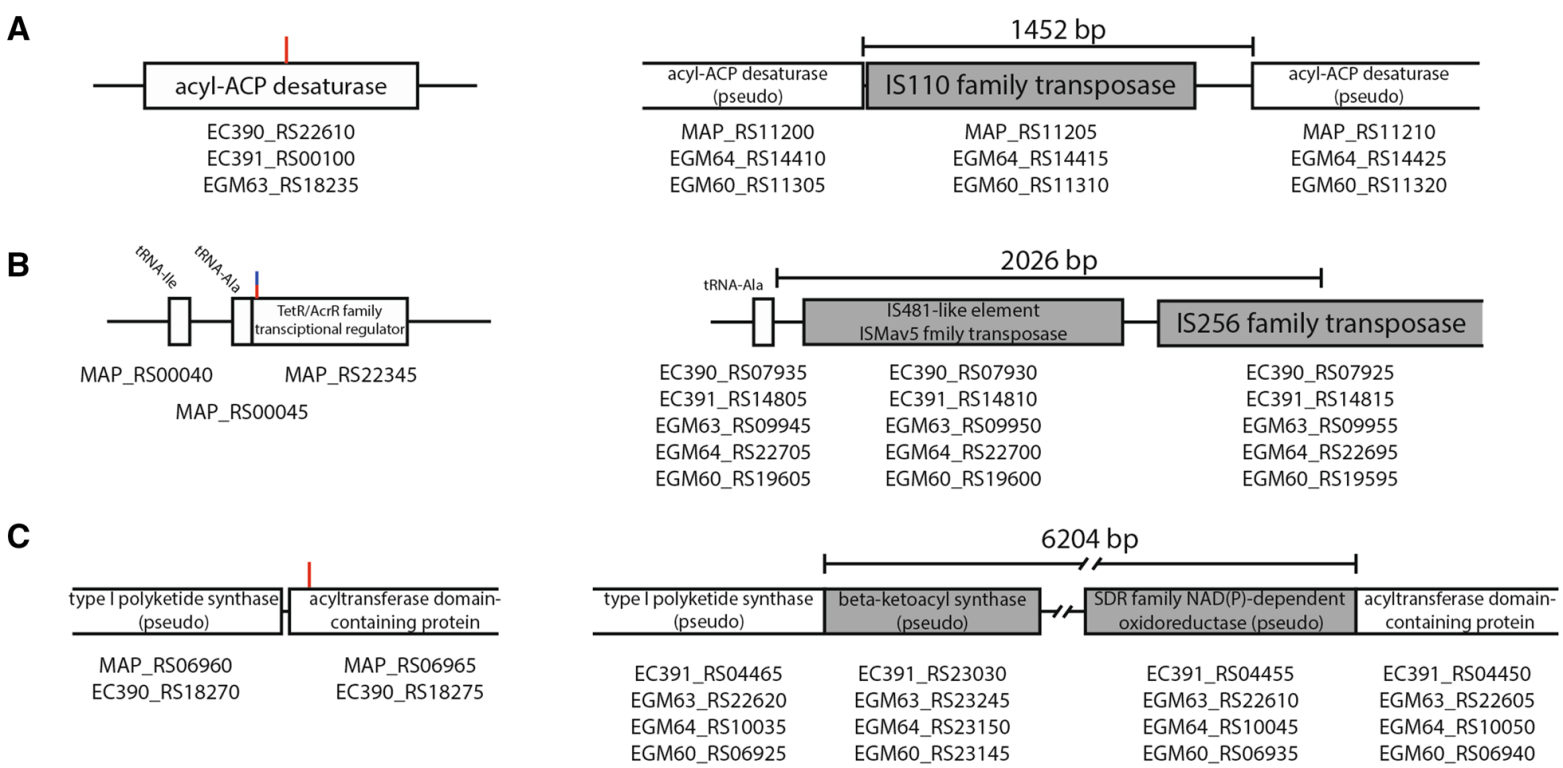
**Detailed information of large gaps (< 10 kb).** **A** A 1452 bp gap was inserted in the middle of the CDS, which was predicted to be acyl-ACP desaturase. **B** A 2026 bp gap was inserted after the gene was predicted to be tRNA-Ala. **C** A 6204 bp gap was inserted on the front side of the CDS, which was predicted to be an acyltransferase domain-containing protein. The red line in each picture indicates the position in which the gap is inserted, and the blue line in **B** indicates the inverting position.

To compare the entire genome of Korean isolates, genome-wide alignment with complete genome sequences was performed using the Mauve tool (Figure 2). The most notable observation from the results was that a certain portion of MAPK genomes were inverted compared to the reference genome K-10. The inverted genome positions were the middle of two genes, IS*256* family transposase (MAP_RS19260) and TetR/AcrR family transcriptional regulator (MAP_RS22345), which were located at approximately 4.2 Mb and 11 kb in K-10. This inverted sequence was also found in other MAP genomes. Two strains (JII-1961 and TANUVAS) showed the same form as K-10, while other C- and B-type MAPs showed inverted forms (Additional file 2). Based on the results of Mauve alignment, gaps were analyzed between K-10 and five MAPK strains to determine the large sequence polymorphisms (LSPs) (Additional file 3). Among the gaps analyzed, three notable gaps (larger than 1000 bp) were observed (Figure 3). The largest gap observed was 6204 bp, which was annotated as a “repeated sequence” from Rapid Annotation using Subsystem Technology (RAST) [21] annotation (Figure 3C). This 6204-bp sequence was present in MAPK_CN7, MAPK_CN4, MAPK_JB16, and MAPK_JJ1 but absent in MAPK_CN9 along with K-10. Depending on the NCBI database, a gap was inserted between two genes, type I polyketide synthase (MAP_RS06960 and EC390_RS18270) and acyltransferase domain-containing protein (MAP_RS06965 and EC390_RS18275), generating two new genes, beta-ketoacyl synthase (EC391_RS23030, EGM63_RS23245, EGM64_RS23150, and EGM60_RS23145) and SDR family NAD(p)-dependent oxidoreductase (EC391_RS04455, EGM63_RS22610, EGM64_RS10045, and EGM60_RS06935), which were annotated as pseudogenes.

The second largest gap measured 2026 bp (Figure 3B). Half of the gap had a similar sequence as IS_*MAP03,* and the other half of the gap had a similar sequence as IS*1311*. The gap was inserted after the tRNA-Ala gene (MAP_RS00045) and generated two additional transposases, IS*481*-like element IS*Mav5* family transposase (EC390_RS07930, EC391_RS14810, EGM63_RS09950, EGM64_RS22700, and EGM60_RS19600) and IS*256* family transposase (EC390_RS11115, EC391_RS11625, EGM63_RS06755, EGM64_RS02850, and EGM60_RS22795). A notable feature of this gap was that its inserted location was the end of the genome inverted region (Figure 2).

The last large gap was a 1452 bp gap, which could be observed in K-10, MAPK_JB16, and MAPK_JJ1 (Figure 3A). The nucleotide sequences of the gap were identified as IS*900* from the NCBI database, and it was found that the gap contained an IS*110* family transposase (MAP_RS11205, EGM64_RS14415, and EGM60_RS11310). This discovery was confirmed by determining that three strains (K-10, MAPK_JB16, and MAPK_JJ1) have 17 conserved regions that have nucleotide sequences similar to those of IS*900,* while the other three strains (MAPK_CN9, MAPK_CN7, and MAPK_CN4) have 16 conserved regions. The gap was inserted into the middle of acyl-ACP desaturase (EC390_RS22610, EC391_RS00100, and EGM63_RS18235), separating it into two pseudogenes (MAP_RS11200, MAP_RS11210, EGM64_RS14410, EGM64_RS14425, EGM60_RS11305, and EGM60_RS11320).

**Figure 4:**
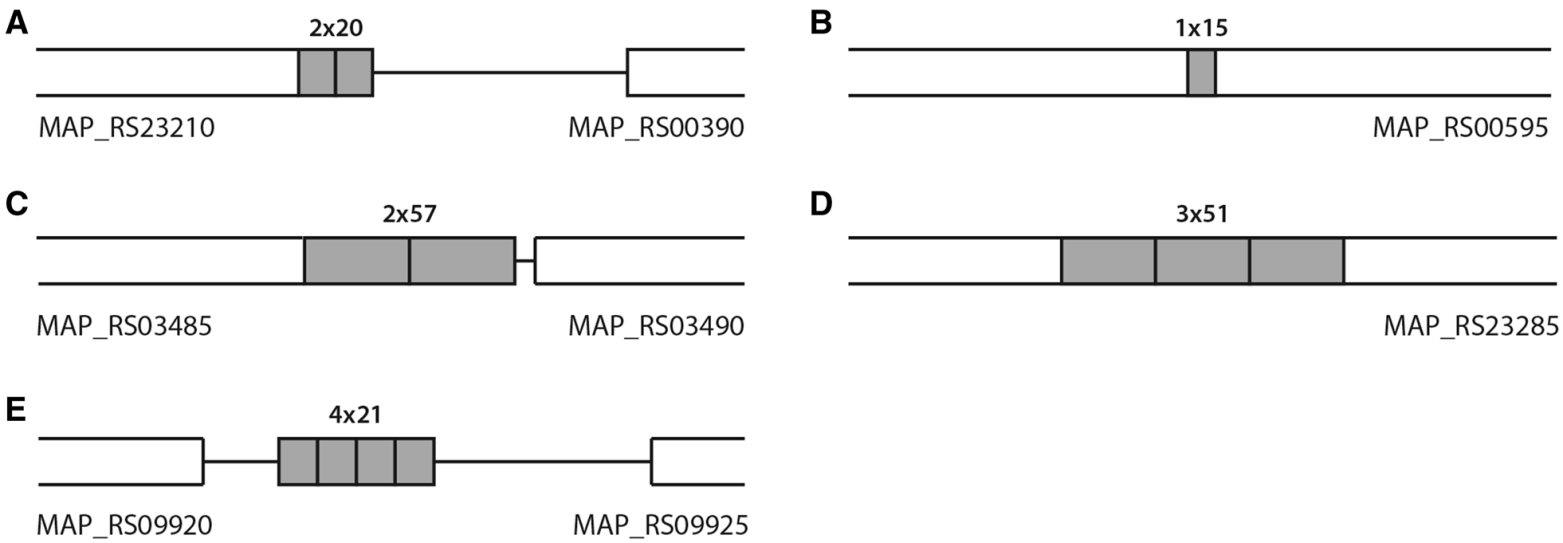
**Schematic representation of novel tandem repeats with reference genome K-10.** **A** MAPK_TR1. **B** MAPK_TR2. **C** MAPK_TR3. **D** MAPK_TR4. **E** MAPK_TR5

Among the smaller gaps found in this study, seven sequences were found to be TRs (Table 1 and Figure 4). Two of these sequences were identical to MIRU292 and VNTR25 [9], and the other five were considered novel TRs. In a previous study, MIRU-VNTR typing discriminated MAPK strains into two types, INMV2 and INMV68, corresponding to the C-type and B-type, respectively [18]. However, in silico typing with the novel TR candidates discriminated the Korean strains into four different types (Table 2). Further in silico typing with 13 complete genomes, including Korean strains, was discriminated into seven different types by the novel TRs (Table 2). Five genomes (E1, E93, FDARRGOS, JII-1961, and TANUVAS) were identical to K-10, whereas strain Telford showed differences compared to K-10 in most targets, except MAPK_TR_2. Each TR discriminated the strains into two (MAPK_TR_1 and 2) or three (MAPK_TR3, 4 and 5) different types. Typing with the new TR has been found to still differentiate the classic type based on the IS*1311* PCR-REA. The discriminatory index (DI) with the novel TRs was calculated as 0.795 among the 13 genomes, whereas the DI value of the original MIRU-VNTR was 0.628. When the eight existing TRs and five novel TRs were considered together, the DI value was calculated as 0.872.

**Table 1:** Detailed information on the discovered tandem repeats during gap analysis

Name of TR	Position of tandem repeats on *M. avium* subsp. *paratuberculosis* (K-10) genome		Length of TR (bp)	Sequence of TR
	Start	Stop		
MAPK_TR_1	83 156	83 195	20	AATTAACGATATCGAATTTG
MAPK_TR_2	127 386	127 400	15	CCGCCGACCAGCTCT
MAPK_TR_3	703 849	703 948	57	ACGACCATTAAACAAGGAGTGATCGCGAGCGCGGGCGAAGCCCGGGTGAAGCGGGTC
MAPK_TR_4	1 798 372	1 798 524	51	CCCGGCGGCGGCGGTGGCAGCATCCCCGGTGGCCCGACCGGTGGCGGCGGC
MAPK_TR_5	2 156 149	2 156 235	21	CGCCGCGCCCGTCGAGCGTCA
MIRU292	3 253 654	3 253 812	53	GTCATCTGCGCCGCTCCTCCTCATCGCTGCGCTCTGCATCGTCGTCGGCGCGA
VNTR25	3 665 698	3 665 855	58	CTCCTGCGCATCCCGCTGCGCGGAATGCTTCGTCGCCGGGCTCCACCCCAATCACCCA

**Table 2:** In silico genotyping of 14 complete MAP genomes by using novel tandem repeats found in this study

	MAPK_TR_1	MAPK_TR_2	MAPK_TR_3	MAPK_TR_4	MAPK_TR_5	TR Type	IS*1311* type
Telford	1	1	1	1	2	1	S
MAPK_JB16	1	1	3	3	3	2	B
MAPK_JJ1	1	1	3	3	3	2	B
MAPK_CN4	1	2	3	3	3	3	B
MAPK_CN9	2	1	1	3	3	4	C
MAPK_CN7	2	1	2	2	3	5	C
MAP4	2	1	2	3	3	6	C
K-10	2	1	2	3	4	7	C
E1	2	1	2	3	4	7	C
E93	2	1	2	3	4	7	C
FDAARGOS_305	2	1	2	3	4	7	C
JII-1961	2	1	2	3	4	7	C
TANUVAS	2	1	2	3	4	7	C

### Comparative and functional characterization of *M. avium* subsp. *paratuberculosis* genomes based on pangenome analysis

**Figure 5:**
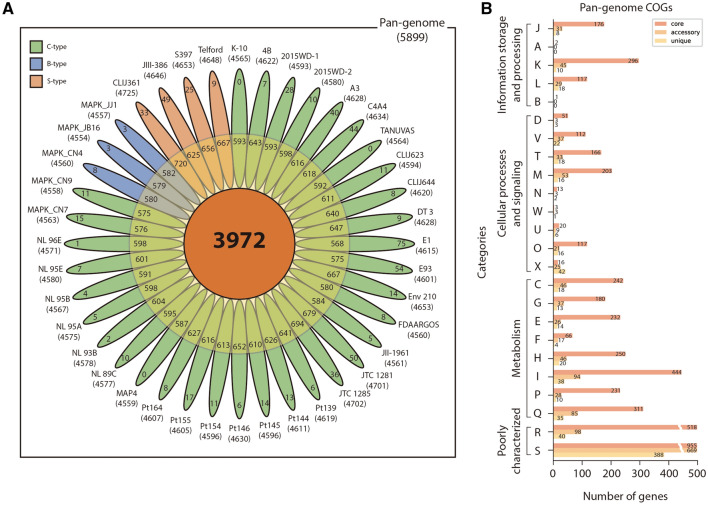
**Pangenome analysis for 5 newly isolated strains and 35 publicly available** ***M. avium*** **subsp.** ***paratuberculosis*** **genomes.** **A** The gene distribution of pangenome. The pangenome is divided into core, accessory, and unique genomes. **B** Each genome is classified into 24 COG categories; J: translation, ribosomal structure and biogenesis, A: RNA processing and modification, K: transcription, L: replication, recombination and repair, B: chromatin structure and dynamics, D: cell cycle control, cell division, chromosome partitioning, V: defense mechanisms, T: signal transduction mechanisms, M: cell wall/membrane/envelope biogenesis, N: cell motility, W: extracellular structures, U: intracellular trafficking, secretion, and vesicular transport, O: posttranslational modification, protein turnover, chaperones, X: mobilome: prophages, transposons, C: energy production and conversion, G: carbohydrate transport and metabolism, E: amino acid transport and metabolism, F: nucleotide transport and metabolism, H: coenzyme transport and metabolism, I: lipid transport and metabolism, P: inorganic ion transport and metabolism, Q: secondary metabolite biosynthesis, transport and catabolism, R: general function prediction, S: function.

Genomic comparison was performed using the 40 MAP whole genome sequences obtained from NCBI (Additional file 4). The genomes were reannotated by the RAST pipeline [21] and analyzed with the BPGA [17] pipeline. The average number of predicted coding sequences (CDSs) was 4918. Over 40 MAP genomes, the number of pangenomes was 5899, including 3972 (67.3%) core genomes, 1268 (21.5%) accessory genomes, and 659 (11.2%) unique genomes (Figure 5A and Additional file 5). It was confirmed that MAP genomes were closed pangenomes by analyzing highly conserved genome sequences and a high ratio of core genomes (Additional file 6). Some accessory genes were identified as type-specific genes (Additional file 7). Among the accessory genes, 139 genes (11% of accessory genome) were shared and only present in S-type genomes, while 56 of them were absent in all S-type genomes. Nine B-type-specific genes were observed. Seven genes were only present in B-type genomes, while two genes were absent. The C-type genes have only one common shared gene. The large number of S-type-specific genes could be new evidence that S-type MAPs are relatively distant from other MAP strains and share similar genes with MAC strains [30].

After the pangenome analysis, functional analysis was conducted with the Clusters of Orthologous Groups (COGs) database [28]. Through COG analysis, each gene was distinguished into 24 categories depending on their roles (Figure 5B, Additional file 5). Among the 24 categories, S (function unknown) occupied the largest part followed by R (general function prediction only). Among well-characterized genes, many of the genes (core genome, 41.5%; accessory genome, 26.4%; unique genes, 20.5%) were mapped into categories related to metabolism. The largest number of genes in the core and accessory genomes (444, 94) belonged to group I (lipid transport and metabolism). In the case of unique genes, the largest number of genes were related to metabolism, especially X (mobilome: prophages and transposons), despite accounting for a smaller portion of the pangenome. From these results, it is presumed that the diversity of MAP has been secured by evolving through the mutation or HGT of genes associated with metabolism, especially lipid metabolism.

### Phylogenetic analysis among 40 *M. avium* subsp. *paratuberculosis* strains

**Figure 6:**
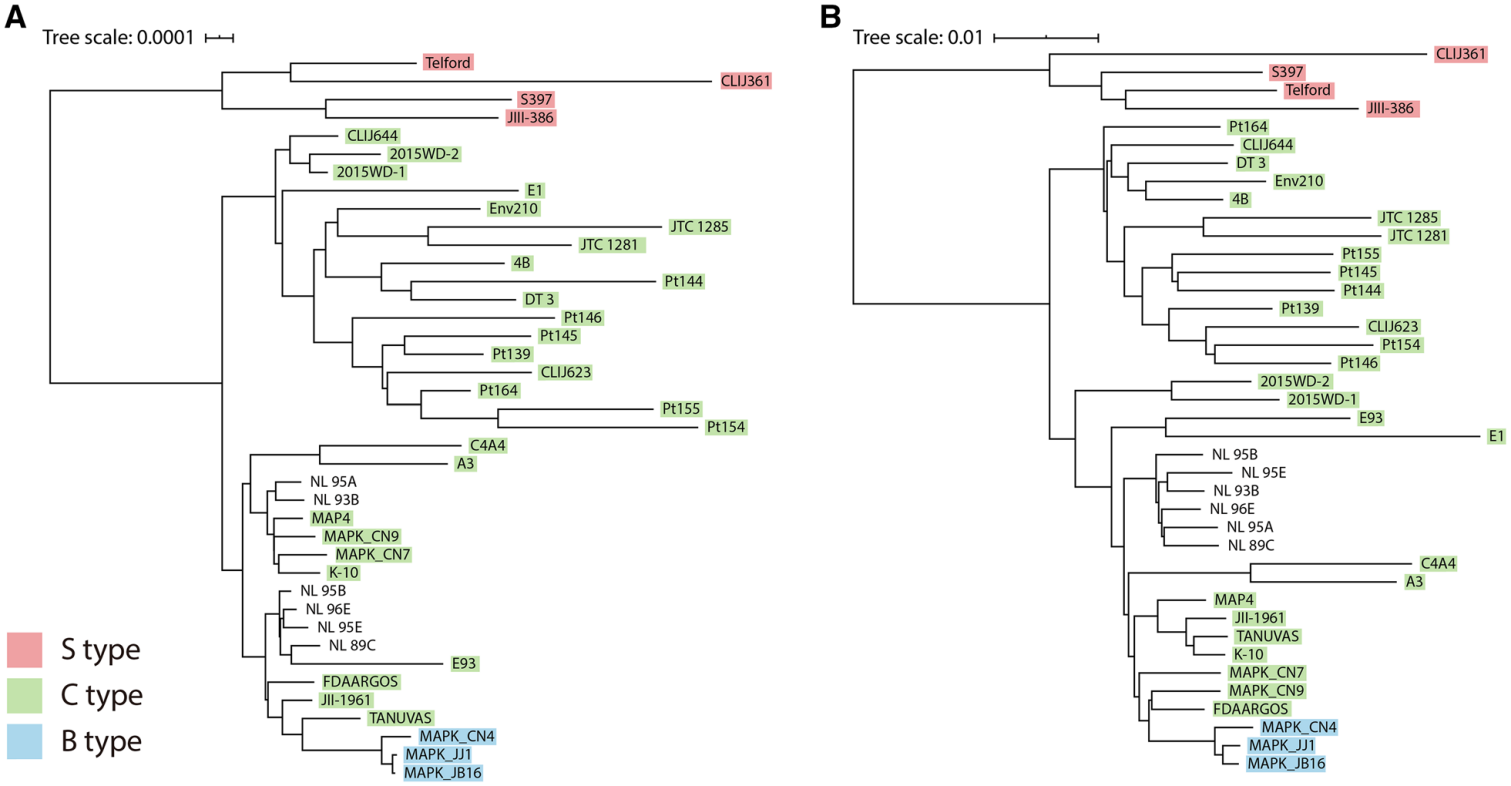
**Phylogenetic relationship between 40 MAP strains.** **A** Core-genome phylogeny based on concatenated core gene alignment. **B** Binary panmatrix phylogeny.

Pangenome-based phylogenetic analysis was performed among 40 MAP strains for the identification of genetic diversity, especially the classification of strains. Major genotypes of MAP have been employed to classify the strains into three types, S-, B-, and C-type, which are distinguished by polymorphisms of the IS*1311* element [4, 11]. A phylogenetic tree was constructed through core phylogeny (Figure 6A) based on concatenated core gene alignment. The phylogenetic tree showed two main clusters representing the S-type and C-type. The tree divided C-type strains into two clusters, and B-type strains were grouped in a cluster. As a result, the B-type is considered to be a subgroup of the C-type, as claimed previously by Bryant et al. [31]. Classification of C-type strains into two clusters was observed by core phylogeny using amino acid sequences (Additional file 8). All strains were classified into the same clusters with the core-phylogeny classification with nucleotide sequences, except E1. From the results, it is suggested that C-type strains could be distinguished into two subgroups. Among S-type strains, there was a subdivision into two branches representing Type I (CLIJ361 and Telford) and Type III (JIII-386 and S397), previously defined by pulsed field gel electrophoresis (PFGE) (Figure 6A) [32].

In the case of the panphylogeny (Figure 6B), a tree based on the binary matrix of the pangenome showed a similar pattern compared with the core phylogeny. There were two main branches dividing C- and S-type strains, and the subdivision of C-type strains was also observed. However, some strains, including 2015WD-1, 2015WD-2, and E1, belonged to different clusters when compared to Figure 6A. Instead, a tendency was observed that strains isolated from the same region formed the same subgroup, except the S-type strains (Additional file 4).

### Analysis of virulence related genes in pangenome

From the results of pangenome analysis, VF-related genes were searched with three different databases, that is, VFDB, Victors, and PATRIC [25–27]. Among the pangenomes, 335 core genomes, 73 accessory genomes, and 40 unique genomes were identified as VFs, excluding the overlapping results (Additional file 9). From the 448 virulence-related genes, type-specific genes and well-known MAP VFs were further analyzed, such as the PE/PPE, *mbt*, *mce*, and *mmp*L genes.

There were 13 type-specific genes that were related to VFs (Table 3). Two clusters, 31 and 23, had 99.2% and 100% identity to the mbtE gene, but cluster 31 was found only in the S-type genomes, while cluster 23 was found in the rest of the genomes. Similarly, clusters 510 and 1384 exhibited similar results. Both genes had similar sequences as *mbt*A, but the former was a gene found only in S-type genomes, while the latter was a gene found in the rest of the genomes. It was reported that the S-type genome has different lengths and sequences in the *mbt*A and *mbt*E genes [30], and it was confirmed that these differences were common features of S-type genomes in this study. Other clusters, such as clusters 442, 1170, 1221, 1841, 2721, 2849, 2857, and 5731, were genes identified only in S-type genomes, and their predicted functions were related to metabolism, which was similar to a previous report [30]. These genes were on large sequence polymorphisms (LSPs) that are only found in the S-type [30, 33].

**Table 3:** S-type specific virulence factor genes

Cluster	Predicted function	Ortholog virulence factor	Database	Reference	Identity (%)	Note
31	Nonribosomal peptide synthetase, partial [WP_019730007.1]	VFG009488(gi:41408271) (mbtE) MbtE [Mycobactin (CVF315)]	VFDB	*Mycobacterium avium* subsp. *paratuberculosis* K-10	99.2	Only in S
442	GMC family oxidoreductase [WP_003874392.1]	fig|83332.12.peg.3806|Rv3409c|VBIMycTub87468_3806| Cholesterol oxidase (EC 1.1.3.6) @ Steroid Delta(5)→Delta(4)-isomerase (EC 5.3.3.1)	PATRIC	*Mycobacterium tuberculosis* H37Rv	87.2	Only in S
510	AMP-binding protein [WP_003875986.1]	VFG009545(gi:118462251) (mbtA) 2,3-dihydroxybenzoate-AMP ligase [Mycobactin (CVF315)]	VFDB	*Mycobacterium avium* 104	99.3	Only in S
1170	Glycosyltransferase [WP_003876129.1]	VFG029573(gi:118465574) (gtf1) glycosyl transferase [GPL locus (CVF650)]	VFDB	*Mycobacterium avium* 104	98.6	Only in S
1221	Glycosyltransferase [WP_003876135.1]	VFG029631(gi:118466342) (gtf2) glycosyltransferase 28 [GPL locus (CVF650)]	VFDB	*Mycobacterium avium* 104	98.8	Only in S
1841	NAD-dependent epimerase/dehydratase family protein [WP_016706194.1]	VFG029546(gi:118464065) (rmlB) NAD dependent epimerase/dehydratase [GPL locus (CVF650)]	VFDB	*Mycobacterium avium* 104	99.7	Only in S
2721	Class I SAM-dependent methyltransferase [WP_031350157.1]	VFG029564(gi:387876472) (rmt4) macrocin-*O*-methyltransferase [GPL locus (CVF650)]	VFDB	*Mycobacterium intracellulare* str. MOTT36Y	90.5	Only in S
2849	Class I SAM-dependent methyltransferase [WP_029245480.1]	VFG029626(gi:523914475) (rmt3) MtfD protein [GPL locus (CVF650)]	VFDB	*Mycobacterium yongonense* 05-1390	92.9	Only in S
2857	Class I SAM-dependent methyltransferase [WP_009977502.1]	VFG029564(gi:387876472) (rmt4) macrocin-*O*-methyltransferase [GPL locus (CVF650)]	VFDB	*Mycobacterium intracellulare* str. MOTT36Y	85.5	Only in S
5731	Acyl carrier protein [WP_019306489.1]	VFG021806(gi:118462750) (MAV_2873) acyl carrier protein [Mycobactin (CVF315)]	VFDB	*Mycobacterium avium* 104	97	Only in S
23	MbtE [AAS04490.1]	VFG009488(gi:41408271) (mbtE) MbtE [Mycobactin (CVF315)]	VFDB	*Mycobacterium avium* subsp. *paratuberculosis* K-10	100	Not in S
1384	AMP-binding protein [WP_003875986.1]	VFG009544(gi:41408276) (mbtA) MbtA [Mycobactin (CVF315)]	VFDB	*Mycobacterium avium* subsp. *paratuberculosis* K-10	100	Not in S

PE/PPE are proteins that are thought to be a source of antigenic and genetic variation, since they have well-conserved N-terminal genes with variable C-termini [34]. Fifty-four genes were predicted as PE/PPE proteins from the pangenome, and among them, 13 genes returned hits from the BLASTp results (Additional file 9). Of the 13 genes, nine belong to the core genome, two belong to the accessory genome, and two belong to the unique genome. Furthermore, there were five type-specific PE/PPE genes that were not detected with BLASTp results. Two genes (clusters 1060 and 3760) were S-type specific genes, another two genes (clusters 169 and 5462) were S-type absent genes, and the other gene (cluster 3973) was a B-type specific gene. Finally, two clusters, 720 and 1200, were predicted as hypothetical proteins with reannotation, but they showed high identity (89% and 100%) to the PPE gene in the VF database. The other clusters predicted by the same function appear to be a problem caused by the poor quality of genomes used in the analysis (Additional file 4).

The *mbt* genes are essential for the survival of MAP to synthesize mycobactin, and they consist of a cluster with ten genes, extending from *mbtA* to *mbtJ* [35]. Twenty-six mbt genes were found in pangenome. In most of the cases, mbt genes were well conserved and well clustered into the core genome (Additional file 9). The MbtA and MbtE genes are type-specific genes already mentioned. Some mbt genes were found in multiple clusters, but they were determined to be similar sequence clusters after scrutiny. Four clusters (33, 38, 67, and 151) returned hits to the *mbtF* gene, three clusters (5151, 5171, and 5234) were identified as *mbtH*, two clusters (62 and 206) were identified as *mbtB*, two clusters (88 and 1991) were identified as *mbtD*, two clusters (671 and 1010) were identified as *mbtI*, and two clusters (3582 and 4183) were identified as *mbtK*. Other *mbt* genes, *mbtC*, *mbtG*, and *mbtJ*, were found to match clusters 1075, 1131, and 2318, respectively.

The mce genes are cell-wall proteins that generally form a cluster consisting of five genes from *mceA* to *mceF* [36–38]. It was reported that the reference MAP genome, K-10, has 8 *mce* gene clusters [39]. The number of genes predicted as *mce*-family proteins was 59 in the pangenome (Additional file 9). Except for cluster 5856, 58 other genes were identified as virulence genes of the *mce* family, and one additional hypothetical protein (cluster 1845) was also returned as a hit to the *mce* gene from the BLASTp results. Three interesting results were obtained through this comparison. First, absence of one mce cluster was observed. It was found that the JIII-386 genome had seven *mce* clusters, while the other genomes had eight [39]. The JIII-386 genome had a defective *mce2* cluster that had only *mceA* and *mceB* genes in the cluster region. Second, type-specific point mutations in certain mce genes were identified. Cluster 1862, which is identified as *mce4B*, had two point mutation positions depending on type. The S-type, B-type, and four C-type (E1, TANUVAS, 2015WD-1, and 2015WD-2) have mutations (Cys → Arg) in the 62^nd^ amino acid sequence. Additionally, S-type genomes had additional mutations (Gly → Val) on the 245^th^ amino acid sequence of the same cluster. Cluster 1955, which was identified as *mce7B*, also had an S-type specific mutation (Lue → Phe) on the 282^nd^ amino acid sequence. Third, CDSs that compose the cluster 850, identified as *mce7D*, consisted of one long CDS in the S-type genome and two sequential short CDSs in other genomes.

The mycobacterial membrane protein large (*mmpL*) proteins are essential genes for physiological and pathogenetic reasons [40, 41]. A total of 23 genes were predicted as *mmpL* in the pangenome, and eight *mmpL* genes were returned as hits from the BLASTp results (Additional file 9). There was one type specific *mmpL* gene. Cluster 111 was a gene that could not be found in all S-type genomes. Four *mmpL* genes, *mmpL4a*, *mmpL4b*, *mmpL10*, and *mmpL11*, were well conserved in all MAP genomes. The *mmpL3* gene was hit with two accessory clusters 99 and 117, but after further analysis, it was confirmed that *mmpL3* was also conserved in all MAP genomes. Furthermore, it appears to be reasonable to combine two clusters (108 and 114) into one group and consider them core genes with *mmpL6* because the protein sequences were very similar. Three clusters (99, 106, and 117) should also be considered as one cluster with the *mmpL3* gene, which is well conserved in all MAP genomes.

### Design and confirmation of *M. avium* subsp. *paratuberculosis* detection biomarker from pangenome analysis

**Figure 7:**
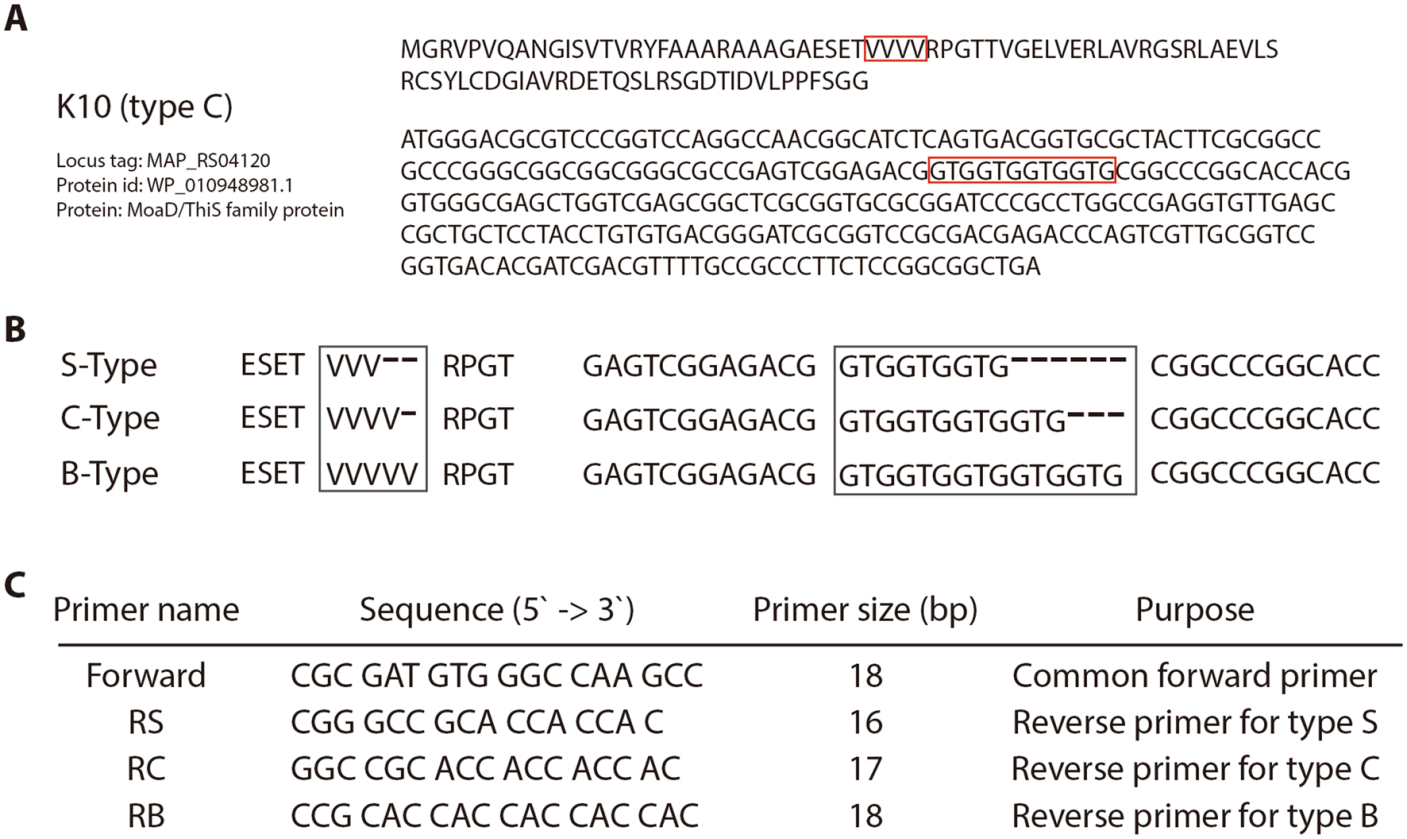
**Novel candidate for genotyping S-, B-, and C-type MAP strains using real-time PCR assay.** **A** DNA and protein sequences of the MoaD/ThiS gene in the reference genome K-10. **B** V (GTG) repeat variation depends on types. **C** Information on PCR primers for genotyping.

Interesting information was observed in three clusters (4950, 4966, and 4977) when searching the accessory genome. The predicted function of the three clusters was the same as that of the MoaD/ThiS family protein (Figure 7A). Many regions of short sequence repeats (SSRs) have been reported [8], but this region is a novel discovery. The gene has a protein sequence with 4 repeats of Val (GTG) from the 32^nd^ to 35^th^ (Figure 7A, red box). This area has a different pattern depending on the type. Their repetition numbers were 3 times in the S-type and 5 times in the B-type (Figure 7B).

For the detection of sequence variation, a polymerase chain reaction (PCR)-based detection method was designed. The genome region 92 bp upstream of the gene was selected as the forward primer binding region, and the reverse primer binding region was selected on the repeated region (repeat of GTG) for differential amplification depending on the type (Figure 7C). Real-time PCR was selected to monitor amplification efficiency in real time because it was expected to show different amplification efficiencies if 3 to 6 gaps were present in the primer against the target template. Therefore, three different primers were amplified with each type of strain to observe amplification efficiency according to its specificity to primers. Fortunately, not only the gene sequence but also the surrounding sequences were well conserved.

**Figure 8:**
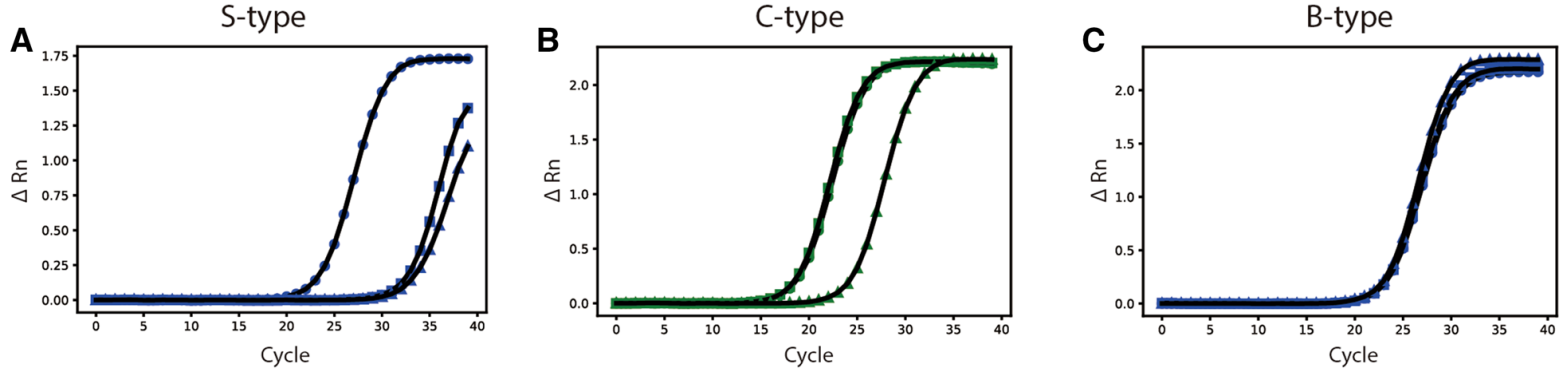
**Amplification plot of newly designed type-discriminating real-time PCR.** **A** Expected result of the S-type. **B** Expected result of the C-type. **C** Expected result of the B-type. Filled circle: S-type primer, filled square: C-type primer, filled triangle: B-type primer.

The expected result was that the S-type strain was specifically amplified only in primer RS, whereas the C-type strain was specifically amplified for primers RS and RC, and the B-type was specifically amplified in all primer sets (Figure 8). Five strains were selected for each C- and B-type. Although S-type strains were not available in this study, the other two *M. avium* strains, *M. avium* subsp. *avium* 101 (MAA 101) and *M. avium* subsp. *hominisuis* 104 (MAH 104), have the same gene as the S-type strain; therefore, two *M. avium* strains were used for PCR verification.

Real-time PCR was successfully performed, and it appears that novel primers can distinguish the type of MAP (Additional file 10). As mentioned above, MAA 101 and MAH 104 were used as alternatives to the S-type and showed that primer RS was amplified first with a Ct value of approximately 24–26, while other primers, RC and RB, were amplified 6–7 cycles later than RS (Figure 8A, Additional file 10). The results for C-type MAP were also clear (Figure 8B, Additional file 10). Both primers, RS and RC, amplified together with similar times (Ct value approximately 20–21), and primer RB was delayed by approximately 5 cycles. Amplification of all three primers in B-type MAP was confirmed (Figure 8C, Additional file 10), and the Ct value was 24–26 in all primers. Other results of the experimental MAP strains are available in Additional file 10. The primers were tested at various annealing temperatures (54–60 °C), and no bias was observed. These findings may support the classification of MAP into S-, C-, and B-types.

## Discussion

*Mycobacterium avium* subsp. *paratuberculosis* (MAP) is an important pathogen in the dairy cattle industry due to its economic impact [1], and it has infected ruminants worldwide [2]. It is important to examine the genetic diversity of the population for epidemiological analysis or to observe various pathogenic properties. As advances in next-generation sequencing (NGS) methods have been made, it has become easier to obtain genomic information from organisms [42], which has led to the rise of bioinformatics [43–45]. Comparative genomics is a powerful method to identify genes that cause phenotypic differences and vice versa [16, 39, 46]. MAP has a highly conserved genome, and it has been difficult to determine its genetic diversity. In this regard, the correlation between genetic diversity and phenotypic differences has primarily focused on the major strain types.

In this study, we performed whole genome sequence (WGS) alignment to determine the genetic diversity of the MAP strains. It was found that there is a chromosomal change in the MAP genomes. All C-type and B-type genomes had partially inverted genomes compared to reference K-10, except TANUVAS and JII-1961. The inversion of the genome could be used as a new criterion to distinguish MAP strains, since they have highly conserved genomes. Another interesting point of genome inversion was that there was a large gap in Korean isolates in the position of the inversion. This gap was one of the large gaps we observed, measuring 2026 bp in size, and it contained a transposase. It was reported that both phenomena, genome inversion and transposition, often result in gene duplication [47, 48]. Thus, a novel transposase was added when the inversion occurred. Another observed large gap was 6204 bp in size. The interesting point of this gap was that it could not be found in reference genome K-10, but it was found in *M. avium* subsp. *hominissuis* (MAH) strains, including strains 104 and TH135, with 100% query coverage and 98.6% and 98.45% identity from the NCBI BLAST results (data not shown). Based on the CDSs in the MAH 104 strain, two proteins, acyltransferase domain-containing protein (MAV_RS14825) and type I polyketide synthase (MAV_RS14830), were annotated in the region covering the 6204-bp sequence. BLAST results of the MAPK_JJ1 and MAH 104 strains showed deletion of two consecutive nucleotide sequences in MAPK_JJ1. This finding might have resulted in the annotation of pseudogenes in MAPK strains. Taken together, these results suggest that a 6204-bp sequence was deleted in strains K-10 and MAPK_CN9.

The genotyping of MAP strains was classified by using the INMV database [9], which discriminated the MAP strains with eight MIRU-VNTRs. We found five new tandem repeat regions in MAP strains and tested their validity in silico. As a result, it may be possible to design a typing method with higher resolution by adding newly discovered TRs to the existing MIRU-VNTR candidates. However, it should also be considered that the novel TRs could discriminate MAPK strains in a wider variety than other strains. A possible reason is that novel TRs were identified when comparing only MAPK and K-10 strains. Therefore, laboratory testing with various MAP strains is needed to determine whether these new candidates are useful for analyzing genetic diversity.

Pangenome analysis was performed to compare the MAP genomes after WGS alignment. Pangenome analysis is a useful tool for comparing genome data to identify the common features and differences of genomes at the same time. Through the analysis, it was confirmed that MAPs have well-conserved genomes and that their pangenomes are closed pangenomes. However, some clusters were formed incompletely. Even though the genome data were filtered before pangenome analysis, 15 of 40 genomes had low quality and had more than 450 contigs (Additional file 4). The number of pangenomes could be further curtailed if all analyzed genomes were complete genomes. To obtain more accurate results, we need more high-quality MAP genomes from various isolation regions.

The distance of MAP genomes was calculated with the pangenome results and represented as phylogenetic trees. The phylogenetic trees were separated into two main branches representing S-type and C-type strains, and B-type strains were positioned in a cluster. In core genome phylogeny, SNPs of conserved genes are the primary targets for analysis, whereas panphylogeny-based trees contain all genes for analysis; therefore, such evolutionary events as HGT can be explained [49]. This finding could support the notion that pangenome-based trees have higher divergence than core genome-based trees [49, 50]. Therefore, pangenome-based analysis can provide basic data to determine transmission because the data could represent regional characteristics of MAP strains. Taken together, the results of pangenome-based phylogenetic analysis suggest the further classification of C-types into two subgroups and provide basic data that enable more detailed epidemiological analysis of MAP strains.

The last task that we performed in this study was identifying a different sequence of the gene depending on the type and designing a real-time PCR method by using a new biomarker to identify the type of MAP strain. From the pangenome analysis, three remarkable genes were identified that had different sequences according to their types. The number of MAP strains we used to test the new marker was 12 (two for S-type, five each for B-type and C-type), which is a notably small number. Even though the number of tested samples was small, PCR experiments were performed under various conditions. Additionally, from this experiment, we confirmed that real-time PCR could be employed to check single nucleotide polymorphisms (SNPs) in the genes. Furthermore, in the same gene, the repeat count of A on the 23^rd^ of the protein was different between types I and III, which are subgroups of S-type. Type I has 3 A repeats, while type III has 2 A repeats. Thus, it would be more efficient to check the whole sequences of genes using sequencing technology.

## Supplementary Information

**Additional file 1.** **Circular visualization of genomic reconstruction for the five MAPK strains.** (A) MAPK_CN4/13. (B) MAPK_CN7/15. (C) MAPK_CN9/15. (D) MAPK_JB16/15. (E) MAPK_JJ1/13.

**Additional file 2.** **Whole genome sequence alignment of 14 complete genomes available in public databases.** Sequence alignment with Mauve software showed that two strains (JII-1961 and TANUVAS) had same form with K-10, while other C- and B-type MAPs had inverted forms.

**Additional file 3.** **Genomic gaps between MAPK strains and the K-10 strain identified by Mauve analysis.**

**Additional file 4.** **List of whole genome sequences analyzed in this study.**

**Additional file 5.** **List of pangenome data.**

**Additional file 6.** **Pangenome and core genome plots analyzed by the Bacterial Pangenome Analysis tool.** Pan and core genome plot showed that MAP genome has almost closed genome.

**Additional file 7.** **Cluster of genes identified as S-, B-, C-type specific.**

**Additional file 8.** **Phylogenetic relationship between 40 MAP strains based on core genome phylogeny with amino acid sequences.** Amino acid sequence-based core genome phylogeny showed similar result with nucleotide-based core genome phylogenetic analysis.

**Additional file 9.** **List of genes mapped to virulence factor databases (VFDB, PATRIC, and Victors).**

**Additional file 10.** **Amplification plot of newly designed type-discriminating real-time PCR using 12 different MAP strains.** Representatives of S-type (M. avium strain 101 and 104) showed only single specific amplification, whereas C-type strains showed double specific amplification. B-type strains were amplified with all three primers.

## Data Availability

All data generated or analyzed during this study are included in this published article.

## Abbreviations

MAP: *Mycobacterium avium* subsp. *paratuberculosis*
MAA: *Mycobacterium avium* subsp. *avium*
MAH: *Mycobacterium avium* subsp. *hominisuis*
SNP: Single nucleotide polymorphism
MIRU-VNTR: Variable-number tandem repeats of mycobacterial interspersed repetitive units
MLSSR: Multilocus short sequence repeat
WGS: Whole genome sequencing
TR: Tandem repeat
VF: Virulence factor
DI: Discriminatory index
CDS: Coding sequence
COG: Clusters of orthologous group
LSP: Large sequence polymorphism
PFGE: Pulsed field gel electrophoresis
SSR: Short sequence repeats
PCR: Polymerase chain reaction
NGS: Next-generation sequencing
