# Supplementary figures and images for: Genomic diversity of Mycobacterium avium subsp. paratuberculosis: pangenomic approach for highlighting unique genomic features with newly constructed complete genomes
Source: Vet Res. 2021 Mar 18;52:46. doi: 10.1186/s13567-021-00905-1 (PMC7977185; doi:10.1186/s13567-021-00905-1)

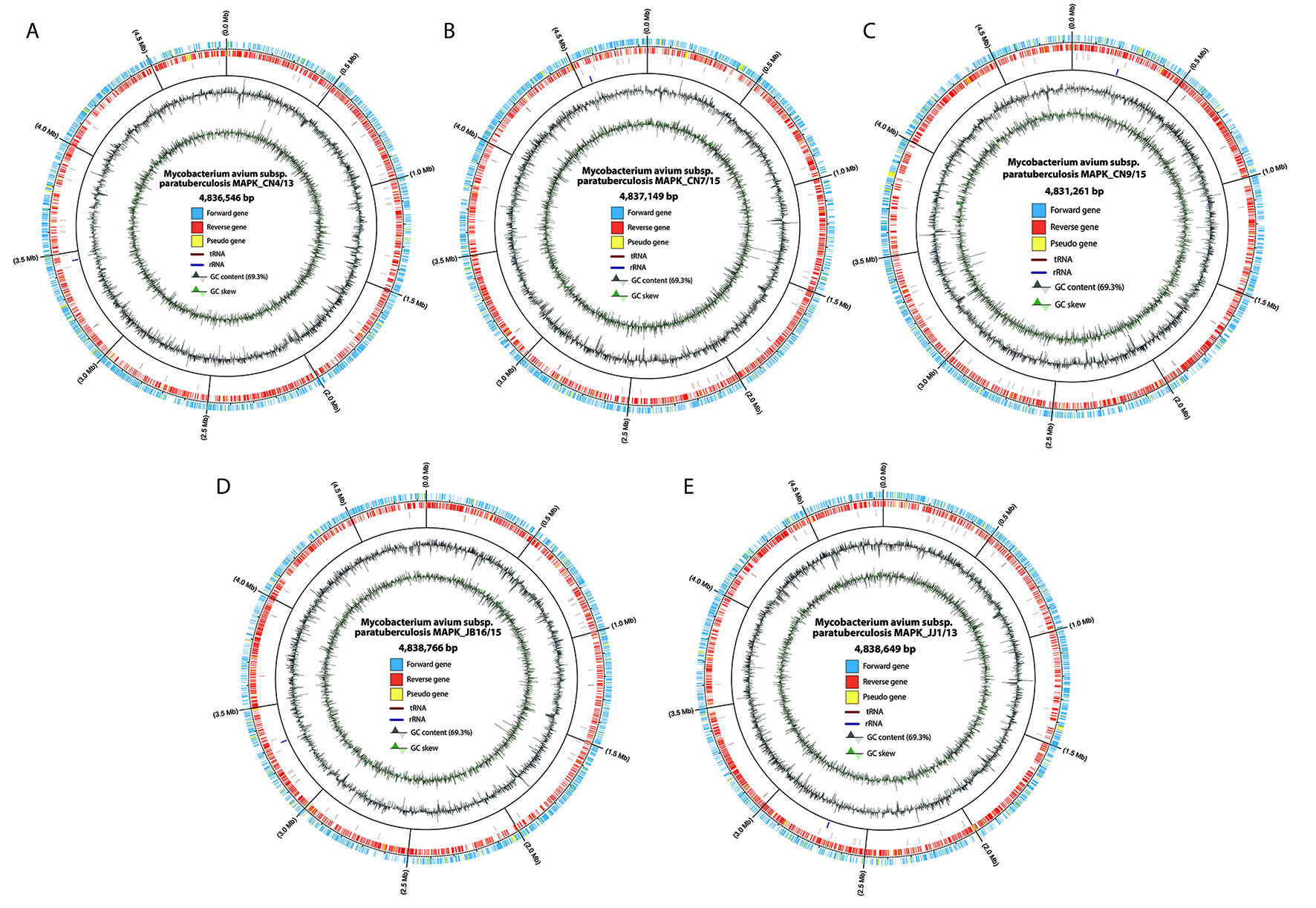

Supplement: Supplementary file 1 — Additional file 1. Circular visualization of genomic reconstruction for the five MAPK strains. (A) MAPK_CN4/13. (B) MAPK_CN7/15. (C) MAPK_CN9/15. (D) MAPK_JB16/15. (E) MAPK_JJ1/13. [file 13567_2021_905_MOESM1_ESM.tif]

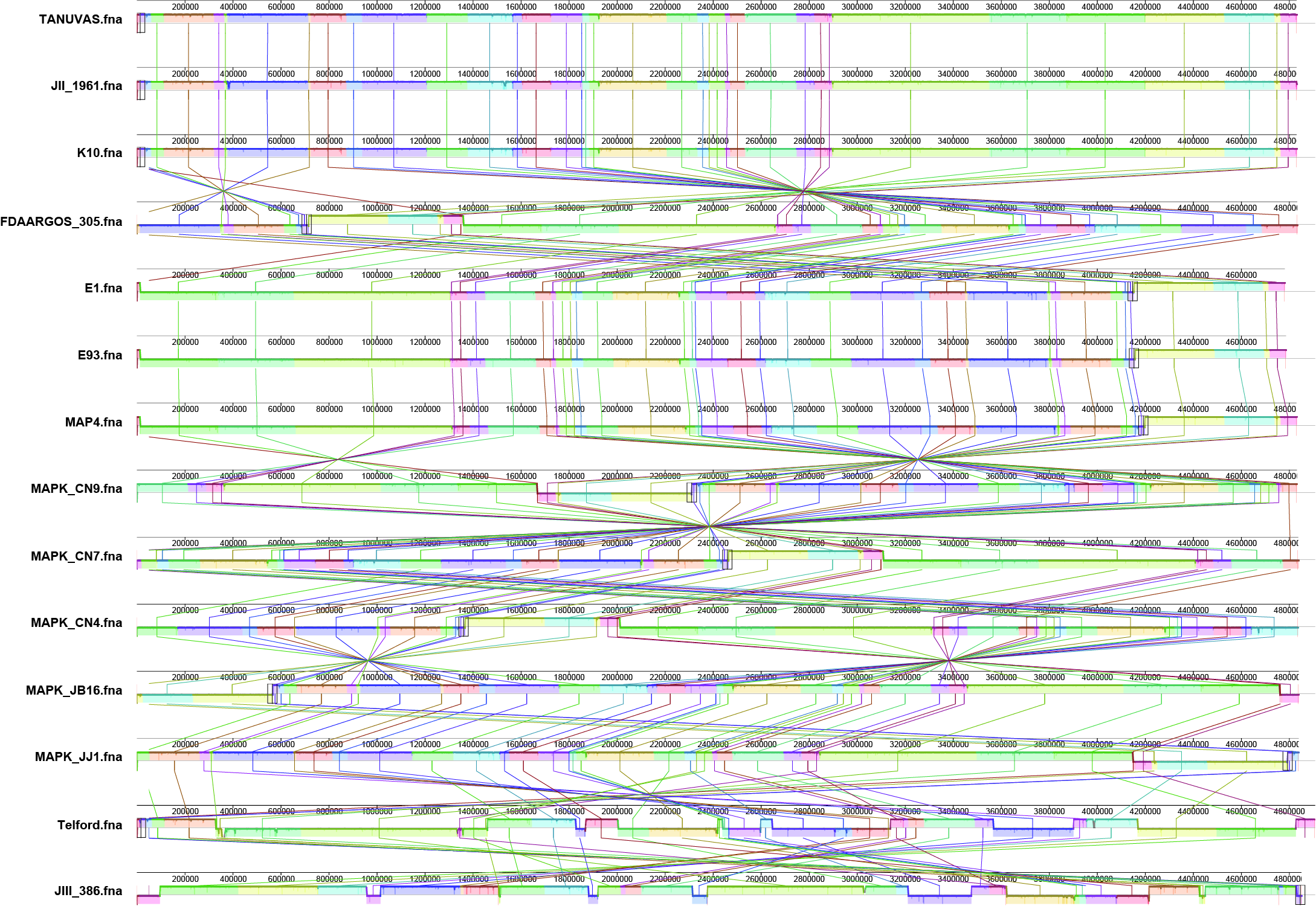

Supplement: Supplementary file 2 — Additional file 2. Whole genome sequence alignment of 14 complete genomes available in public databases. Sequence alignment with Mauve software showed that two strains (JII-1961 and TANUVAS) had same form with K-10, while other C- and B-type MAPs had inverted forms. [file 13567_2021_905_MOESM2_ESM.tif]

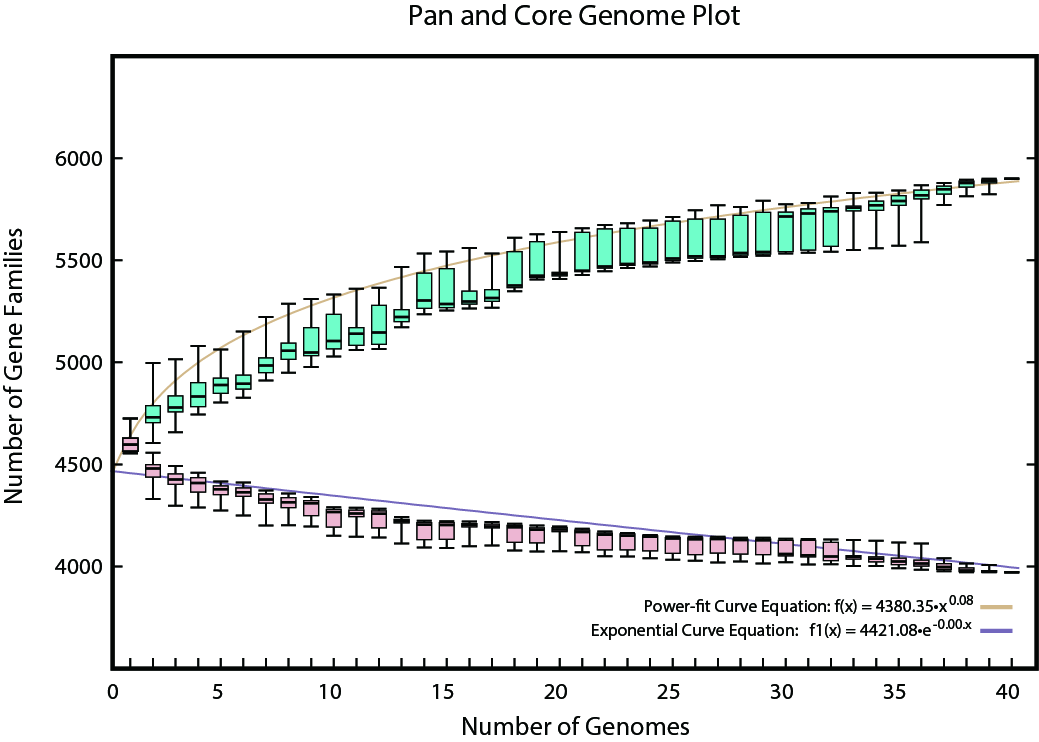

Supplement: Supplementary file 6 — Additional file 6. Pangenome and core genome plots analyzed by the Bacterial Pangenome Analysis tool. Pan and core genome plot showed that MAP genome has almost closed genome. [file 13567_2021_905_MOESM6_ESM.tif]

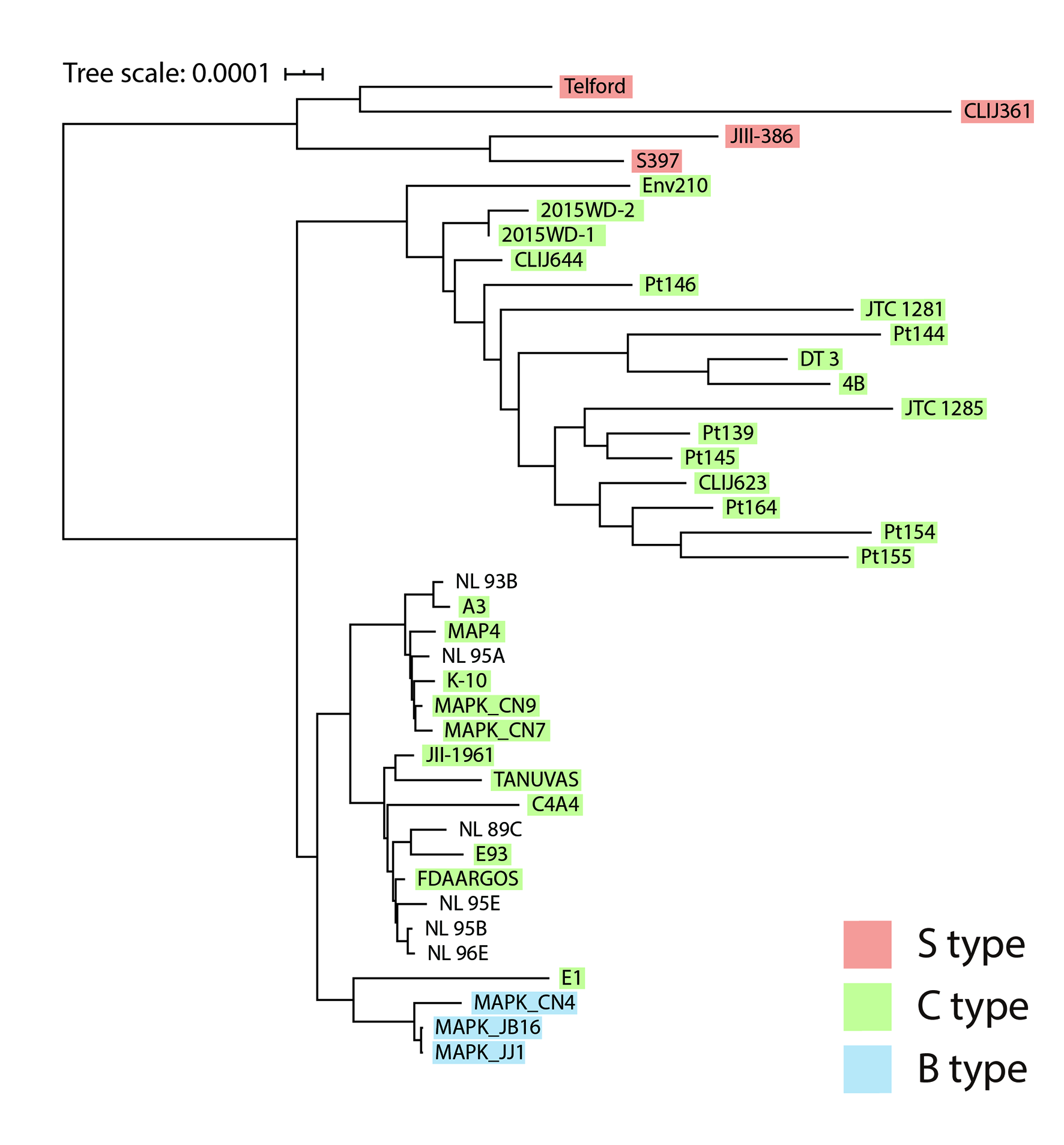

Supplement: Supplementary file 8 — Additional file 8. Phylogenetic relationship between 40 MAP strains based on core genome phylogeny with amino acid sequences. Amino acid sequence-based core genome phylogeny showed similar result with nucleotide-based core genome phylogenetic analysis. [file 13567_2021_905_MOESM8_ESM.tif]

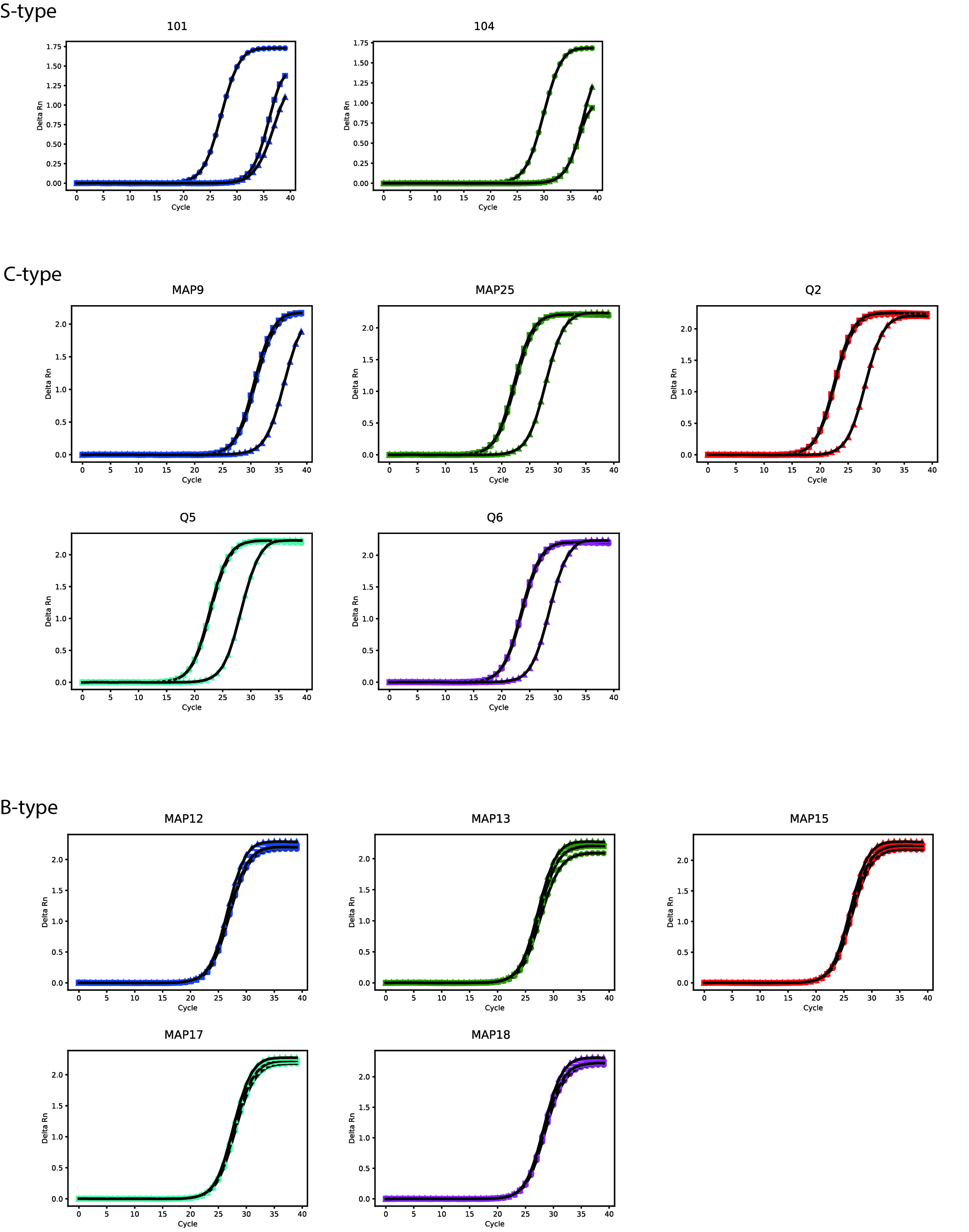

Supplement: Supplementary file 10 — Additional file 10. Amplification plot of newly designed type-discriminating real-time PCR using 12 different MAP strains. Representatives of S-type (M. avium strain 101 and 104) showed only single specific amplification, whereas C-type strains showed double specific amplification. B-type strains were amplified with all three primers. [file 13567_2021_905_MOESM10_ESM.tif]
